# The relationship between allergic diseases and neurodevelopmental disorders in children enrolled in primary healthcare

**DOI:** 10.12669/pjms.41.2.10691

**Published:** 2025-02

**Authors:** Yusuf Adnan Guclu, Erdem Erkoyun, Selcuk Sinan Celik, Demet Can

**Affiliations:** 1 Yusuf Adnan Guclu, MD. Associate Professor, Department of Family Medicine, Izmir Faculty of Medicine, Health Sciences University, Tepecik Training and Research Hospital, Izmir, Turkiye; 2 Erdem Erkoyun, MD. Specialist, Public Health Services Presidency, Provincial Health Directorate, Izmir, Turkiye; 3 Selcuk Sinan Celik, MD. Specialist Pediatric Clinic, Health Sciences University, Dr. Behçet Uz Children’s Diseases, Surgery Training and Research Hospital, Izmir, Turkiye; 4 Demet Can, MD. Professor, Department of Pediatric Immunology and Allergy Diseases, Izmir Faculty of Medicine, Health Sciences University, Dr. Behçet Uz Children’s Diseases, Surgery Training and Research Hospital, Izmir, Turkiye

**Keywords:** Autism spectrum disorder, Attention deficit and hyperactivity disorder, Asthma, allergic rhinitis, Atopic dermatitis, Primary healthcare

## Abstract

**Background and Objective:**

The prevalence of neurodevelopmental disorders (NDs) and allergic diseases(ADs) has increased in recent years. This study aimed to analyse the association between them among children registered at primary healthcare units in Izmir.

**Methods:**

For this retrospective cross-sectional study, primary healthcare units in Izmir were selected by randomisation conducted between January 1, 2023 and December 31, 2023. The dependent variables are attention deficit hyperactivity disorder (ADHD) and autism spectrum disorder (ASD). The risk factors are respiratory allergies and skin allergies. Logistic regression models were adjusted for age, sex, socioeconomic level.

**Results:**

There were 20,557 children registered in 26 primary healthcare units. Skin allergy was detected in 7.8%, respiratory allergy in 6.8%, ADHD in 1.2% and ASD in 0.0%. ADHD comorbidity was found to be high in both children with respiratory allergies and children with skin allergies (p<0.001). The rate of ASD comorbidity was 1.8% in boys with respiratory allergies and 1.6% in boys with skin allergies (p>0.01). The analysis could not be performed due to the low number of girls with ASD. The logistic regression models showed a higher odds ratio for respiratory allergies (OR:6.2, 95% CI: 4.6-8.3) than for skin allergies (OR:2.2, 95% CI: 1.5-3.2) for ADHD.

**Conclusion:**

It would be beneficial to increase awareness among healthcare providers and parents about the co-occurrence of NDs and ADs.

## INTRODUCTION

The increase in the prevalence and burden of allergic diseases (ADs) in recent decades has parallelled the worldwide increase in the diagnoses of neurodevelopmental disorders (NDs).^1^ ADs are a significant contributor to the burden of disease in children, and respiratory and skin allergies are the two most important diseases.^2^ The Global Burden of Disease Study estimated the prevalence of asthma at 5.3% and atopic dermatitis at 4.8%.^3^

NDs are a heterogeneous group of disorders that appear early in life and often persist throughout life. Common ND diagnoses include ASD and ADHD. Due to the epidemiological transition, NDs have increased in recent years but are less common than allergic disorders (ADHD 2.8% and ASD 0.4%).^3^

The parallel increase in prevalence suggests that relatively more common ADs may be risk factors for NDs. However, many studies investigating the relationship between ADs and NDs have yielded controversial results.^4^ Although the exact aetiology of these diseases is unknown, interactions of neurobiological, genetic and environmental factors are thought to contribute to the development of the diseases.^5^-^7^ A study conducted in Taiwan showed that children diagnosed with AD under the age of three had a higher risk of developing ADHD or ASD in later childhood. Physicians caring for toddlers with AD should be aware of the increased risk of developing ADHD or ASD later in life, especially if children have comorbidities such as allergic rhinitis, allergic conjunctivitis or asthma.^8^ Studies have indicated that the prognosis of ASD and ADHD is more favourable in cases where early diagnosis and treatment are employed.^9^,^10^

The role of the family physician is to provide both therapeutic and preventive health services to individuals and to offer free primary health care services to all residents in Turkiye. As family physicians are in regular contact with children and their families, they are able to obtain accurate information about the children’s health status.^11^ In this study we aimed to analyze the association between (ADHD and ASD) in primary healthcare units in Izmir.

## METHODS

The present cross-sectional study was conducted between January 1, 2023 and December 31, 2023. First, family health centres (FHCs) in Izmir were randomly selected to participate in the study. However, only 26 of the selected FHCs agreed to take part. There were 20,557 children registered in the FHCs. Each FHC was visited by an author, who retrieved the patient’s file from the electronic medical record. All registered children were included in the study.

### Identification of children with NDs:

Children diagnosed with ICD-10 codes F84.0, F84.1, F84.8, or F84.9 were classified as patients with ASD. Children diagnosed with ICD-10 codes F90.0, F90.1, F90.8, or F90.9 were classified as patients with ADHD.

### Identification of children with allergic diseases:

To diagnose an AD, it was necessary for the same ICD code to appear twice in the child’s file. Asthma and/or allergic rhinitis were classified as respiratory allergies. Atopic dermatitis, urticaria, and angioedema were classified as cutaneous allergies. Potential confounding factors were considered in the analysis, including sex (girls, boys), child age at the last visit, and socioeconomic status. Inferences regarding socioeconomic status were determined according to the Townsend deprivation index.^12^

### Statistical Analysis:

The relationship between categorical variables and dependent variables was examined using the Chi-Square test or Fisher’s exact test. Univariate and multivariate logistic regression models were constructed to assess the individual or combined effects of age, sex, and socioeconomic status. An alpha level lower than 0.05 was accepted as significant. Xxx Hospital Ethical Board approved the study protocol (24/11/2022-774, dated November 24, 2024).

### Informed consent:

The data of this observational study were collected from family physicians who were contacted as key informants; thus, individual patients were not subject to data collection process. As a results, and with the approval of the ethical board, informed consent was not required.

## RESULTS

There are 12,020 girls (58.5%) and 8,537 boys (41.5%) register with FHCs. The most frequent group was 5-9 years olds (5,690, 27,7%). There were 1,591 cases with skin allergies (7.8%), 1,390 with respiratory allergies (6.8%), 256 cases with ADHD (1.2%), and 61 cases with ASD (Table-I).

**Table-I:** Characteristics of children registered to family physicians.

	n	%
Girl	12,020	58.5
Boy	8,537	41.5
0-4 years old	4,601	22.4
5-9 years old	5,690	27.7
10-14 years old	5,637	274
15-18 years old	4,629	22.5
Autism Spectrum Disorder	61	0.3
Attention Deficit Hyperactivity Disorder	256	1.2
Skin allergies	1,591	7.7
Respiratory allergies	1,390	6.8

Chi-square tests in contingency tables showed significant associations between ADs and NDs. ADHD was detected in 9.1% of boys with respiratory allergies, and this was statistically significant (p<0.001). A total of 17 cases (2.8%) of ADHD were reported in girls with respiratory allergies, (p < 0.001). Among boys with skin allergies, ADHD was detected in 4.4% of cases, (p < 0.001). Among girls with skin allergies, ADHD was detected in 1.3% of cases, (p < 0.005).

The prevalence of ASD in boys with respiratory allergies was found to be 1.8%, (p>0.001). Because only seven cases of ASD in girls were reported, it was not possible to observe a significant relationship between ADs and ASD. There was a significant relationship with ASD (1.6%) in boys with skin allergies (p<0.001). In girls, this relationship is not significant (0.1%) (Table-II).

**Table-II:** Association of allergic diseases with neurodevelopmental disorders by sex.

		ADHD		ASD		Total

		Yes	No	Yes	No	
Boys with Respiratory Allergies	Yes	72 (9.1%)	719 (91.0%)	14 (1.8%)	777 (98.0%)	791 (100.0%)
	No	124 (1.6%)	7,622 (98.0%)	40 (0.5%)	7,706 (99.0%)	7,746 (100.0%)
	Total	196 (2.3%)	8,341 (98.0%)	54 (0.6%)	8,483 (99.0%)	8,537 (100.0%)
	Pearson Chi-Square Test: p < 0.001			Pearson Chi-Square Test: p < 0.001		
Girls with Respiratory Allergies	Yes	17 (2.8%)	582 (97.2%)	0 (0.0%)	599 (100.0%)	599 (100.0%)
	No	43 (0.4%)	11,378 (99.6%)	7 (<0.1%)	11,414 (99.9%)	11,421 (100.0%)
	Total	60 (0.5%)	11,960 (99.5%)	7 (<0.1%)	12,013 (99.9%)	12,020 (100.0%)
	Fisher’s Exact Test: p < 0.001			Fisher’s Exact Test: p > 0.9		
Boys with Skin Allergies	Yes	36 (4.4%)	773 (95.6%)	13 (1.6%)	796 (98.4%)	809 (100.0%)
	No	160 (2.1%)	7,568 (97.9%)	41 (0.5%)	7,687 (99.5%)	7,728 (100.0%)
	Total	196 (2.3%)	8,341 (97.7%)	54 (0.6%)	8,483 (99.4%)	8,537 (100.0%)
	Pearson Chi-Square Test: p < 0.001			Pearson Chi-Square Test: p < 0.001		
Girls with Skin Allergies	Yes	10 (1.3%)	772 (98.7%)	1 (0.1%)	781 (99.9%)	782 (100.0%)
	No	50 (0.4%)	11,188 (99.6%)	6 (<0.1%)	11,232 (99.9%)	11,238 (100.0%)
	Total	60 (0.5%)	11,960 (99.5%)	7 (<0.1%)	12,013 (99.9%)	12,020 (100.0%)
	Fisher’s Exact Test: p = 0.005			Fisher’s Exact Test: p = 0.4		

ADHD: Attention-deficit/hyperactivity disorder; ASD: Autism spectrum disorder.

Logistic regression models were deemed appropriate for boys but could not be applied to girls due to low number of ASD cases. In logistic regression analysis, the risk of ADHD (OR: 6.2, 95% CI: 4.6-8.3) is high in those with respiratory allergies. Despite adjustments for age and socioeconomic status, the risk of ADHD in those with respiratory allergies remained elevated, with overlapping confidence intervals (Table-III).

**Table-III:** Examination of the Co-occurrence of Respiratory and Skin Allergies with ADHD and ASD in Boys with Logistic Regression

Risk factor	Odds ratio (95% confidence interval)			

	Univariate	Age	Socio-economic development	Age and socio-economic development
***Attention-Deficit/Hyperactivity Disorder***				
Respiratory Allergy	6.2 (4.6-8.3)	5.1 (3.7-6.9)	5.9 (4.3-7.9)	4.8 (3.5-6.6)
Skin Allergy	2.2 (1.5-3.2)	1.9 (1.3-2.7)	2.7 (1.8-3.9)	2.4 (1.6-3.5)
***Autism Spectrum Disorder***				
Respiratory Allergy	3.5 (1.9-11.4)	3.0 (1.6-9.6)	-	-
Skin Allergy	3.1 (1.2-5.7)	2.6 (1.0-4.9)	-	-

In univariate analysis, a 2.2-fold increase (95% CI: 1.5-3.2) in the risk of skin allergies was observed. This risk decreased when adjusted for age (OR: 1.9, 95% CI: 1.3-2.7), and increased when adjusted for socioeconomic deprivation (OR: 2.7, 95% CI: 1.8-3.9). When adjusted for both variables, it remained higher than in univariate analysis (OR: 2.4, 95% CI: 1.6-3.5) (Table-III). Age-adjusted data are provided for boys with ASD only. The risk of ASD is high in boys with both respiratory and skin allergies. When adjusted for age, a reduction in risk is observed compared with univariate analysis (for respiratory allergies: from OR 3.5 (95% CI: 1.9-11.4) to 3.02 (95% CI: 1.6-9.6), for skin allergies: from 3.1 (95% CI: 1.2-5.7) to 2.6 (95% CI: 1.0-4.9)) (Table-III).

## DISCUSSION

This study found that the risk of ADHD and ASD increased in children with respiratory and skin allergies who were registered with FHCs. This risk in allergic children persists for boys, even when adjusted for age and socioeconomic status for ADHD, and for age for ASD. Logistic regression analysis could not be performed due to the insufficient number of cases in girls.

In our study, respiratory allergies were identified in 6.8% of the population, while skin allergies were observed in 7.7%. In the International Study of Asthma and Allergies in Childhood Phase-3 study, asthma (14.1%), allergic rhinitis (14.6%) and eczema (7.3%) were found in the 13-14 age group, while asthma (11.7%), allergic rhinitis (8.5%) and eczema (7.9%) were found in the 6-7 age group.^13^

The American Psychiatry Association reports the prevalence of ADHD in school-age children is approximately 5%. It is worth noting that a wide variability in prevalence rates has been found across studies, ranging from 1% to as high as 20% in school-aged children.^14^ In Turkey, the ADHD prevalence rate was 8%.^15^ In the USA, in children aged 4 and 8 years, the prevalence of ASD is 1.70% and 1.85%, respectively, while in Europe, it varies between 0.38% and 1.55%.^16^

ADHD and ASD have a low prevalence. These rates can be attributed to reasons such as social exclusion. Families may choose to seek help from private health institutions to keep their children’s NDs away from official records.^17^ The study revealed that (ASD) is six times more prevalent in boys than in girls, while (ADHD) was is 4.6 times more common. The prevalence of respiratory allergies in boys was 1.85 times higher and the incidence of skin allergies was 1.45 times higher. The prevalence of ADHD in Pakistan has been found to be around 2.49%. Boys with ADHD outnumber girls, but the ratio varies significantly from 2:1 to 9:1.^18^ Reports on the association between the prevalence of ADs and NDs are inconsistent in the literature. A study conducted in Taiwan examined 46,647 patients with ADs and found an association between ADs with ASD and ADHD.^19^

Nemet and colleagues found that ADHD and ASD were more prevalent in boys. ADHD was found in 23.5% boys with AD and 14.4% of girls. ASD was found in 1.7% of boys with AD and in 0.4% of girls.^4^ In our study, the relationship between ASD and ADHD was more pronounced in boys.

The relationships between NDs and ADs in children are still controversial because these diseases vary depending on age and gender.^20^ However, in this study, adjustments were made for age and socioeconomic status and the relationship was found to continue. Atopic dermatitis is similar to ADHD and ASD in terms of pathogenesis, which includes neuroinflammation and genetics. Data evidence shows significant associations between AD with ADHD and ASD.

In this study, the prevalence of ADHD was slightly lower in the skin allergy group. Children with asthma are at risk of depression and anxiety and growing evidence suggest they may also be at risk of ADHD and ASD. In Kaas et al. ’s meta-analysis, a significant association was found between asthma and ADHD (OR 1.52, 95% CI: 1.42-1.63); however, no association was found between asthma and ASD (OR 1.12, 95% CI: 0.93-1.34).^21^Some of the studies investigating the association between ADs and NDs focused on ASD. A study of 77,951 children in the United States found a positive correlation between ASD and ADs. The prevalence of asthma in children with ASD was over 35%.^22^

In this study, it was determined that socioeconomic status (poverty) was not a significant factor influencing the relationship between allergic conditions and ADHD and ASD. A study conducted by Hammer et al. in 2014 with 9,720 children found no correlation between household income and allergic conditions.^23^

The results of our study have clinical applications for physicians caring for pediatric patients. A strength of this research is that the data were obtained from family physicians, who are responsible for preventive and curative health services. Our study provides solid evidence supporting the association between ADs with ADHD and ASD. ADHD and ASD are more likely in children with respiratory and skin allergies. This risk remains significant in boys, even after controlling for potential confounding factors. This suggests that gender may be a factor in the relationships between ADs and NDs, with a higher prevalence of ADHD and ASD in boys with ADs. The large sample size and analyses considering factors such as gender, age and socioeconomic status make the results more reliable and help us understand these relationships better. This study contributes to the existing literature on the links between ADs and NDs. The study’s strength lies in its use of legal records and randomisation, which yielded a large sample size. We have demonstrated an association between ADs and NDs in a large population. Physicians can diagnose NDs earlier if they consider ADs when evaluating children.

### Limitations:

Despite their limitations in diagnosing ADs or NDs in registered patients, family physicians are able to access the follow-up of their registered populations in secondary or tertiary health centres. Nevertheless, patients in Turkey are permitted to submit direct applications for secondary and tertiary healthcare services. In this study, the role of gender was given particular emphasis in the examination of the relationship between ADs and NDs (ADHD and ASD). However, due to the insufficient number of cases in girls, logistic regression analyses were limited to boys. This makes it challenging to fully comprehend the interrelationship between ADs and NDs in girls, which constrains the generalisability of the findings to the entire population. This deficiency highlights the necessity for future studies with larger sample groups to conduct a more detailed examination of the subjects in question, specifically focusing on female participants.

## CONCLUSION

This study is the first to investigate the association between ADs and NDs in primary healthcare institutions in Türkiye. The study found a significant increase in the co-morbidity of ADHD, ASD among children with ADs. The findings have implications for aetiologic research that searches for common early life antecedents of NDs and ADs. Family physicians, who are often the first healthcare providers that children encounter, can carefully examine children with ADs for ADHD and ASD, facilitating to early diagnosis of these conditions. Findings from this study also should also raise awareness among health care providers and parents about the possible co-occurrence of both NDs and NDs.

### Suggestion:

It is recommended that physicians consider the possibility of ASD and ADHD in individuals with allergic conditions and receive the necessary training to diagnose and treat these conditions effectively. It is imperative that larger cohort studies comprising sufficient numbers of individuals from both genders be conducted.

### Authors’ Contribution:

**YAG, EE, SSC** and **DC:** Conceptualization.

**YAG** and **EE:** Data curation, software.

**YAG, EE** and **SSC:** Formal analysis, writing.

**YAG, EE, SSC** and **DC:** Review & editing.

All authors reviewed the results and approved the final version of the manuscript and are responsible and accountable for the accuracy or integrity of the work.
